# Exercise May Promote Skeletal Muscle Hypertrophy via Enhancing Leucine-Sensing: Preliminary Evidence

**DOI:** 10.3389/fphys.2021.741038

**Published:** 2021-09-24

**Authors:** Yan Zhao, Jason Cholewa, Huayu Shang, Yueqin Yang, Xiaomin Ding, Shaosheng Liu, Zhi Xia, Nelo Eidy Zanchi, Qianjin Wang

**Affiliations:** ^1^Exercise Physiology and Biochemistry Laboratory, College of Physical Education and Health, Wenzhou University, Wenzhou, China; ^2^Exercise Physiology and Biochemistry Laboratory, College of Physical Education, Jinggangshan University, Ji'an, China; ^3^Department of Exercise Physiology, University of Lynchburg, Lynchburg, VA, United States; ^4^School of Sport Medicine and Health, Chengdu Sport University, Chengdu, China; ^5^Hubei Provincial Collaborative Innovation Center for Exercise and Health Promotion, College of Health Science, Wuhan Sports University, Wuhan, China; ^6^Department of Physical Education, Federal University of Maranhão (UFMA), São Luís, Brazil; ^7^Laboratory of Skeletal Muscle Biology and Human Strength Performance (LABFORCEH), São Luís, Brazil

**Keywords:** resistance exercise, aerobic exercise, leucine-sensing, muscle protein synthesis, muscle hypertrophy

## Abstract

Several studies have indicated a positive effect of exercise (especially resistance exercise) on the mTOR signaling that control muscle protein synthesis and muscle remodeling. However, the relationship between exercise, mTOR activation and leucine-sensing requires further clarification. Two month old Sprague-Dawley rats were subjected to aerobic exercise (treadmill running at 20 m/min, 6° incline for 60 min) and resistance exercise (incremental ladder climbing) for 4 weeks. The gastrocnemius muscles were removed for determination of muscle fibers diameter, cross-sectional area (CSA), protein concentration and proteins involved in muscle leucine-sensing and protein synthesis. The results show that 4 weeks of resistance exercise increased the diameter and CSA of gastrocnemius muscle fibers, protein concentration, the phosphorylation of mTOR (Ser2448), 4E-BP1(Thr37/46), p70S6K (Thr389), and the expression of LeuRS, while aerobic exercise just led to a significant increase in protein concentration and the phosphorylation of 4E-BP1(Thr37/46). Moreover, no difference was found for Sestrin2 expression between groups. The current study shows resistance exercise, but not aerobic exercise, may increase muscle protein synthesis and protein deposition, and induces muscle hypertrophy through LeuRS/mTOR signaling pathway. However, further studies are still warranted to clarify the exact effects of vary intensities and durations of aerobic exercise training.

## Introduction

Age-related Sarcopenia (ArS) is a phenomenon of skeletal muscle atrophy and functional decline with advancing age (Bauer et al., 2019). ArS increases the risk of falls, disability and mortality in the elderly, affects both physical and mental health and quality of life, and is a direct contributor to rising healthcare costs in the elderly population (Xia et al., 2017a; Seol et al., 2020). Skeletal muscle anabolic resistance, the dampened muscle protein synthesis response to protein ingestion and mechanical tension, has currently been identified as a major cause of ArS, to which blunted nutrition-sensing contributes (Zhao et al., 2021). Moreover, age-related anabolic resistance was considered to be exacerbated during musculoskeletal disuse and reduced physical activity (Marshall et al., 2020).

The impaired intracellular induction or propagation of mammalian target of rapamycin complex 1 (mTORC1) signaling is a hallmark of anabolic resistance, and thought to be partially a result of weakened anabolic nutrient sensing (Wilkinson et al., 2018). Rag GTPase is the mediator of the mTORC1 pathway in response to nutrient stimulation, specifically the proteogenic amino acid leucine. Four Rag GTPases (A, B, C, and D) are expressed in skeletal muscle. RagA-RagC and RagB-RagD, respectively, from heterodimers to regulate mTORC1 activation induced by different amino acids (Sancak et al., 2008; Lee et al., 2018). In response to leucine stimulation, mTORC1 translocates from the cytoplasm to the surface of lysosomes, interacting with the active heterodimer formed by GTP-loaded RagB and GDP-loaded RagD, and then activates its downstream molecular events (Sancak et al., 2008, 2010). Although mTORC1 signaling is highly sensitive to changes in leucine levels, mTOR itself is not a leucine sensor and cannot detect leucine levels in skeletal muscle cells. Thus, in the process of promoting skeletal muscle protein synthesis and inducing adaptive hypertrophy mediated by mTORC1 in response to anabolic stimulation, a leucine sensor must be involved as a rate-limiting factor. But which proteins are involved as leucine sensors in skeletal muscle, and how they participate in regulation is not fully understood (Lushchak et al., 2019).

To date, Leucyl-tRNA synthetase (LeuRS) and Sestrin2 have been identified as the leucine sensors, both of which are able to regulate mTORC1 signaling (Han et al., 2012; Peng et al., 2014). Among them, LeuRS directly combines with GTP bound RagD and stimulates the GTP hydrolysis of GTPase-activating protein (GAP), and the inactive RagD^GTP^-RagB^GDP^ heterodimer is transformed to a pre-activated state (i.e., RagD^GDP^-RagB^GDP^). Further, the Ragulator complex mediates the nucleotide exchange of RagB as the guanine nucleotide exchange factors (GEF) and forms the active heterodimer RagD^GDP^-RagB^GTP^ which induces the activation and lysosomal translocation of mTORC1. In contrast to LeuRS, Sestrin2 negatively regulates mTORC1 activity by controlling GTP hydrolysis of RagB. Since the Michaelis constant (Km value) of LeuRS for leucine in the amino acids activation reaction and the dissociation constant (Kd value) of leucine for Sestrin2 are similar, whether LeuRS and Sestrin2 co-regulate mTORC1 collectively or separately (Pang and Martinis, 2009; Wolfson et al., 2016) requires further research (Lee et al., 2018). We recently proposed that exercise may effectively enhance the sensitivity of skeletal muscle tissues to amino acid stimulation (Zhao et al., 2021). Resistance exercise has been widely recognized as a potent intervention to maintain or increase skeletal muscle protein synthesis and hypertrophy. Although not considered the most efficient intervention to stimulate a high magnitude of muscle hypertrophy, aerobic exercise can also attenuate the inhibition of muscle protein synthesis and deposition (Xia et al., 2016). However, to date there are no studies that have investigated the role of exercise on the LeuRS/Sestrin2-mTOR axis, and it's not clear which mode of training is most efficacious in regulating mTORC1 signaling and muscle hypertrophy mediated by LeuRS and/or Sestrin2 (Zhao et al., 2021). Thus, the preliminary evidence exploring these relationships are warranted, and the results will inspire more translational research on the topic.

The objective of this study was to preliminarily investigate whether exercise stimulates skeletal muscle mTORC1 signaling via enhancing leucine-sensing (i.e., LeuRS/Sestrin2-mTOR axis), and if an increase in mTORC1 signaling results in skeletal muscle hypertrophy.

## Materials and Methods

### Animals

Thirty-six Sprague-Dawley male rats (200–220 g) were obtained from the Chengdu Dashuo Biological Technology Company (Chengdu, China), and were housed in standard cages in the Laboratory Animal Center of Chengdu Sport University (Chengdu, China). Food (normal rat chow) and water were available *ad libitum* on a conventional 12-h light and 12-h dark cycle. After adaptive feeding for 1 week, the rats were randomly assigned to three groups with twelve mice per group: the sedentary control group (SC), aerobic exercise-trained group (AE), and resistance exercise-trained group (RE). Animal welfare and experimental procedures were conducted according to the Guide for the Care and Use of Laboratory Animals, and the research was approved by the Animal Ethics Committee of Jinggangshan University and Chengdu Sport University. Details with relation to the rats and chow diet used in the present research have been shown in Table 1.

**Table 1:** Details with relation to the rats and chow diet used in this research.

	**SC**	**AE**	**RE**
Age on arrival (week)	~9	~9	~9
Age on sacrifice (week)	~14	~14	~14
Initial body weight (g)	225.8 ± 11.9	220.2 ± 8.2	223.3 ± 10.3
Body weight post-intervention (g)	396.2 ± 26.2	329.5 ± 24.1^**^	348.7 ± 13.3^*^
Gastrocnemius wet weight (g)	2.62 ± 0.12	2.65 ± 0.11	2.73 ± 0.11^*^
Water in chow diet (g/kg)	94	94	94
Crude protein in chow diet (g/kg)	190	190	190
Crude fat in chow diet (g/kg)	51	51	51
Crude fiber in chow diet (g/kg)	36	36	36
Ash in chow diet (g/kg)	62	62	62
Calcium in chow diet (g/kg)	11.3	11.3	11.3
Phosphorus in chow diet (g/kg)	8.6	8.6	8.6

* *P < 0.05 vs. the SC group*;

** *P < 0.01 vs. the SC group*.

### Exercise Training

Rats in the AE and RE groups received 4 weeks (5 days/weeks) exercise training. In brief, the AE rats were subjected to a 3-d accommodative training on a motor-driven treadmill at an estimated 50% VO2max at 0° incline, 10 m/min for 5 min on the first day, 55% Vo2max at 15 m/min for 10 min on the second day, and 60% Vo2max 20 m/min for 10 min on the third day. Thereafter, they were forced to run at a speed of 20 m/min for 60 min (6° incline), which corresponds to an aerobic intensity of ~65% VO2max (Bedford et al., 1979; Qin et al., 2020). In our previous research (Cholewa et al., 2014), we had suggested researchers use the ladder climb model to study hypertrophy or molecular signaling and evaluate samples from muscles with a higher proportion of type II fibers, such as the gastrocnemius. Thus, the ladder climb model (1-m ladder with 2-cm grid steps and inclined at 85°) adapted from Hornerberger et al. was used in the present study (5 days/week) (Hornberger and Farrar, 2004). In line with our previous report (Li et al., 2019), rats were acclimatized to ladder climbing training for 3 days, and the maximal load carrying capacity of each rat was determined. During this determination, rats were initially forced to climb with overload corresponding to 75% bodyweight with compressed air or tactile stimuli on tail, then an additional 30 g load was added until they failed to accomplish the climbing exercise. The maximal workload was record and readjusted each week according to the bodyweight of the rats. In the formal training regimen, rats climbed with the progressively increased workload corresponding to 50, 75, 90, and 100% maximal load carrying capacity, and kept climbing with the 100% load until they cannot accomplish the exercise in spite of tactile stimuli, but, were allowed a 2 min rest every time they reached the top of the ladder.

### Sample Collection

Forty-eight hours after the final training bout (overnight food deprivation), all rats were euthanized with intra-peritoneal injection of 80 mg/kg pentobarbital sodium. Gastrocnemius muscles were separated and washed with PBS, and then were dried by using filter paper. After that, muscles were immediately frozen in liquid nitrogen and stored at −80°C until analysis. Specimens from the contralateral gastrocnemius were fixed for morphological analysis.

### Morphological Examination

Morphological examination was conducted according to our previous study (Xia et al., 2016). In brief, the specimens were fixed in 4% paraformaldehyde solution and embedded in paraffin. Five μm cross-sections were cut from the middle region of the muscles at an axial distance of 120 μm and then subjected to a standard hematoxylin and eosin staining. The cross-sectional area (CSA) and diameter of muscle fibers in each specimen were analyzed using an Olympus DP73 microscope equipped with a Charge-coupled device camera (2,448 × 1,920 pixels) and cellSens Entry image analysis software (Olympus, Tokyo, Japan).

### Myofibrillar Protein Concentration

Myofibrillar fraction from gastrocnemius muscle was obtained by the method described earlier by our group (Xia et al., 2017b). In brief, the muscle samples were homogenized in a 5% ice-cold buffer containing 0.25 M sucrose, 2 mM EDTA, and 10 mM Tris-HCl (pH 7.4). The homogenate was centrifuged at 600 g, then the pellet containing myofibrillar protein was collected. Protein concentration of myofibrillar extract was determined by the BCA (Beyotime, Beijing, China) assay.

### Western Blotting

Western blotting analysis was performed based on the protocol described in our previous study (Xia et al., 2017b; Shang et al., 2019). Briefly, proteins were extracted from muscle specimens and suspended in RIPA buffer supplemented with a Halt protease inhibitor cocktail (Thermo Scientific, Rockford, IL, USA), and protein concentrations were determined. A total of 20 μg of protein samples was loaded per lane, separated via 8 or 12% SDS-PAGE gels and transferred to 0.45 μm nitrocellulose membranes. Membranes were blocked in 3% BSA-TBST at room temperature followed by overnight incubation with primary antibodies at 4°C: 4E-BP1 (1: 2000; 9452s, CST, Beverly, MA, USA), phospho-4E-BP1 (Thr37/46) (1: 1000; 2855s, CST), p70S6K (Thr389) (1: 2000; 9202s, CST), phospho-p70S6K (Thr389) (1: 500; 9205s, CST), mTOR (1: 1000; 2972s, CST), phospho-mTOR (Ser2448) (1: 1000; 2281s, CST), LeuRS (1: 1000; 13868s, CST), Sestrin2 (1: 1000; Ab178518, Abcam, Cambridge, UK), and GAPDH (1: 20000; YM3029, Immunoway, Plano, TX, USA). After incubating the membranes with HRP conjugated secondary antibodies goat anti-mouse or anti-rabbit IgG (1: 10000, 7076 and 7074, CST). The signals were analyzed by using Totallab (Non-linear Dynamics, Newcastle, UK).

### Statistical Analysis

We calculated the required sample size prior to experimentation. When α, power and effect size were set at 0.05, 0.99, and 0.80, respectively, using G^*^power software version 3.1.9.2 (Kiel University, Kiel, Germany), the sample size needed was 36. Normality of the data was checked out and subsequently confirmed using the Shapiro-Wilk test, data that were not normally distributed were transformed and retested. Following transformations, data that failed to meet the assumptions of normality were analyzed with non-parametric tests. Homogeneity of variances was tested using Levene's test for equality of variances, when Levene's test was significant, the Welch ANOVA and Games-Howell pairwise comparison test were used. When the assumptions of normality and homogeneity of variances were both met, the variables were analyzed by one-way ANOVA, and multiple comparison between groups was performed using the Bonferroni *post-hoc* test. All tests were performed using Statistical Package for Social Sciences version 20.0 (SPSS, Inc., Chicago, IL, USA), and all figures were created with Sigmaplot version 12.0 (Systat, San Jose, CA, USA). The effect size were calculated with Cohen's d by using the software G^*^Power. In accordance with Cohen, effect sizes were classified as small (0.2), medium (0.5), or large (0.8). For all analyses, the *P* value of 0.05 or less was considered statistically significant. Data were expressed as means ± standard deviation.

## Results

### Body Weight and Gastrocnemius Wet Weight

After 4 weeks intervention, the body weight of rats in AE (*P* < 0.01) and RE (*P* < 0.05) groups were significantly less than that of SC group. In terms of the gastrocnemius wet weight, rats in RE group were greater than the SC group (*P* < 0.05) (Table 1).

### Morphological Changes of Muscle Fibers

**Figure 1:**
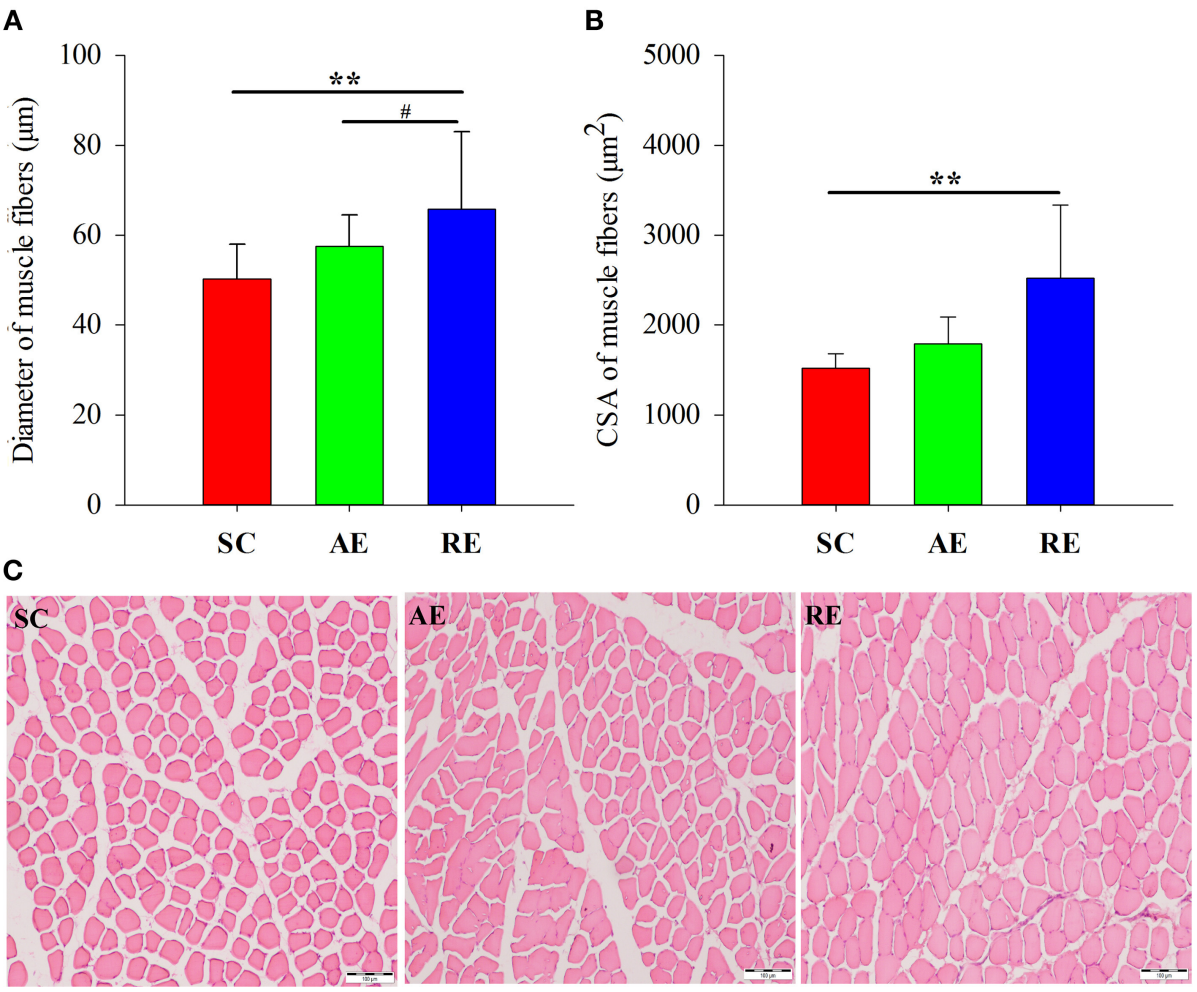
Effects of exercise on **(A)** cross-sectional area (CSA), **(B)** diameter and **(C)** hematoxylin-eosin stained sections of gastrocnemius muscle in rats. Magnification 100×, scale bars = 100 μm. Values are provided as mean ± standard deviation for each group. SC, sedentary control group; AE: aerobic exercise training group; RE, resistance training exercise group. ^**^P < 0.01 vs. the SC group; ^#^*P* < 0.05 vs. the AE group.

The differences of CSA and diameter between groups were statistically significant (CSA: *F* = 5.294, *P* < 0.05; diameter: *F* = 12.423, *P* < 0.01). As for the multiple comparison between groups, the CSA (*P* < 0.01) and diameter (*P* < 0.01) of RE group were significantly greater than the gastrocnemius of SC group, and the diameter were greater than the AE group (*P* < 0.05); however, between AE and SC groups, no statistical significance could be detected (Figure 1).

### Total and Myofibrillar Protein Concentration in Gastrocnemius Muscle

**Figure 2:**
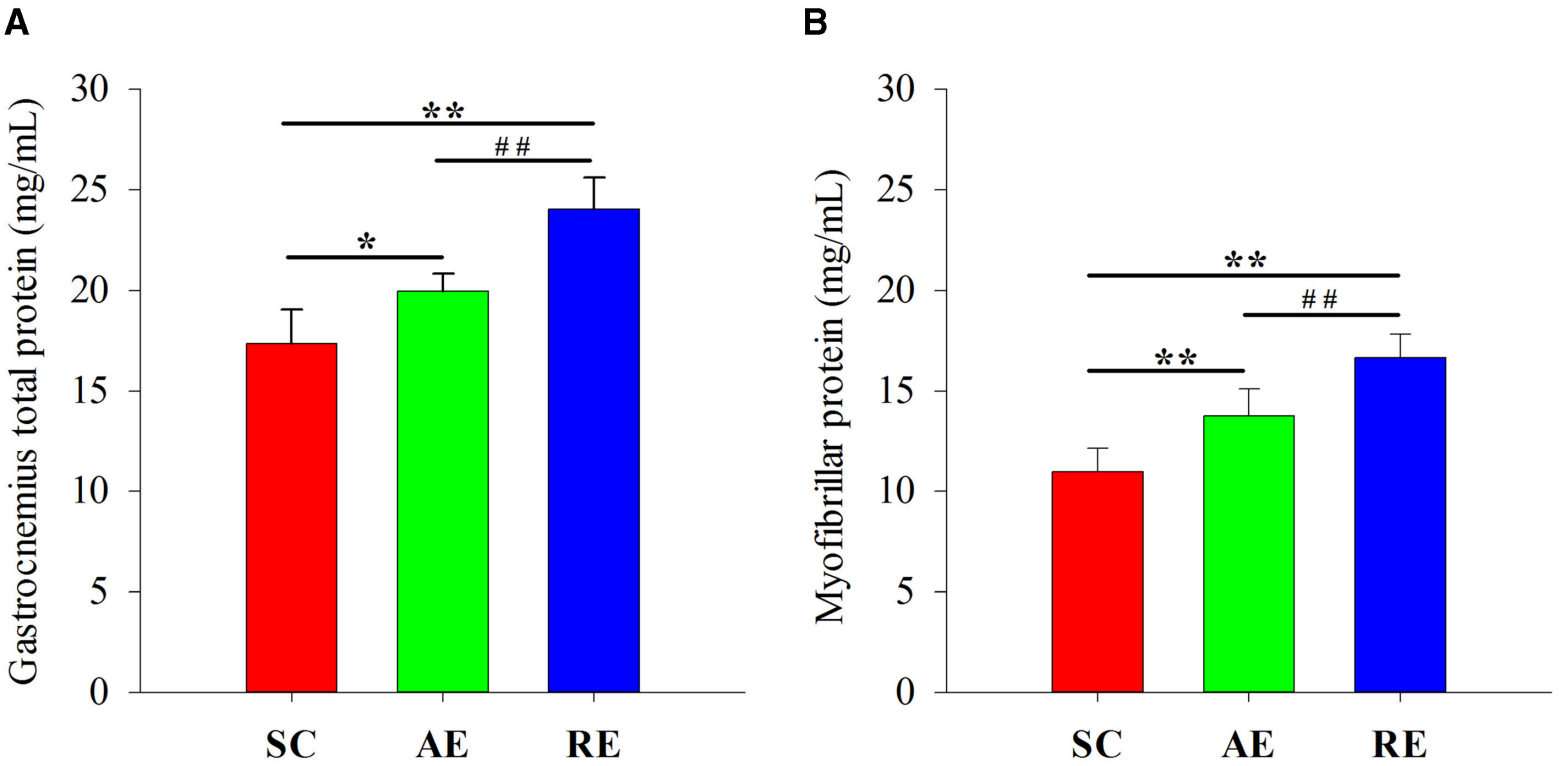
Effects of exercise on **(A)** total and **(B)** myofibrillar protein concentration in gastrocnemius muscle. Values are provided as mean ± standard deviation for each group. SC, sedentary control group; AE, aerobic exercise training group; RE, resistance training exercise group. ^*^P < 0.05 vs. the SC group; ^**^P < 0.01 vs. the SC group; ^##^*P* < 0.01 vs. the AE group.

The difference of total (*F* = 34.106, *P* < 0.01) and myofibrillar (*F* = 31.775, *P* < 0.01) protein between groups were statistically significant. When compared with SC, the concentration of gastrocnemius muscle protein in AE (total: *P* < 0.05; myo: *P* < 0.01) and RE (total: *P* < 0.01; myo: *P* < 0.01) group were significantly greater. The protein concentration in RE was also greater than AE (total: *P* < 0.01; myo: *P* < 0.01) (Figure 2).

### Protein Expression With Relation to Leucine-Sensing

**Figure 3:**
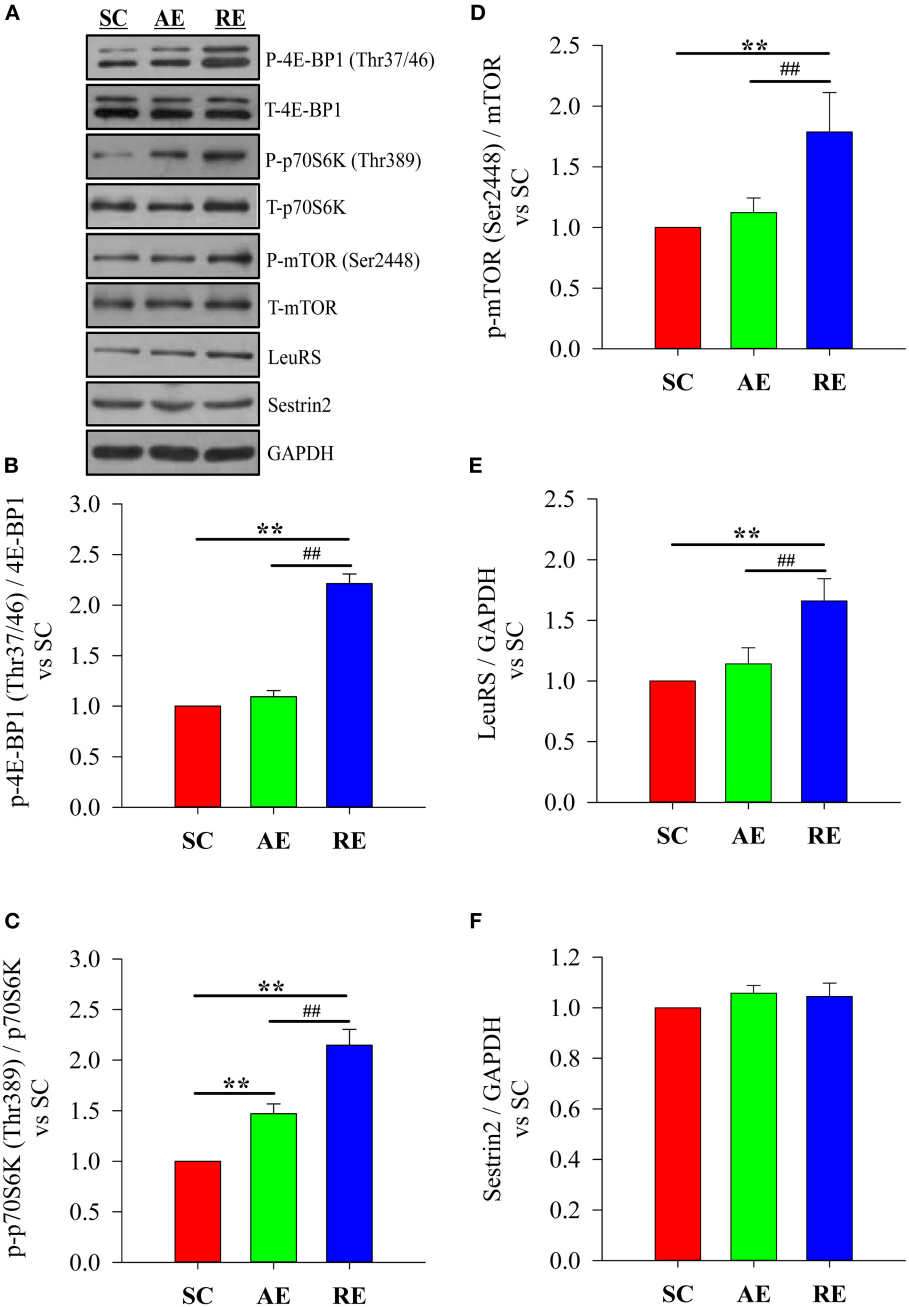
Effects of exercise on protein expression with relation to leucine-sensing. **(A)** blotting, **(B)** phosphorylation state of 4E-BP1 (Thr37/46), **(C)** phosphorylation state of p70S6K (Thr389), **(D)** phosphorylation state of mTOR (Ser2448), **(E)** relative expression of LeuRS and **(F)** relative expression of Sestrin2. SC, sedentary control group; AE, aerobic exercise training group; RE, resistance training exercise group. Data were expressed relative to the SC group. The expression of LeuRS and Sestrin2 were normalized to GAPDH. The phosphorylation state of mTOR signaling was calculated as the ratio of phosphorylated protein to total. ^**^P < 0.01 vs. the SC group; ^##^*P* < 0.01 vs. the AE group.

The results showed that the difference of LeuRS (*F* = 23.084, *P* < 0.01) expression level and the phosphorylation state ratio of 4E-BP1 (Thr37/46) (*F* = 326.493, *P* < 0.01), p70S6K (Thr389) (*F* = 88.649, *P* < 0.01), and mTOR (Ser2448) (*F* = 14.856, *P* < 0.01) between groups were statistically significant, while Sestrin2 (*F* = 2.163, *P* > 0.05) was not. When compared with SC, only the significantly higher phosphorylation state ratio of p70S6K (*P* < 0.01) was detected in AE group; while the LeuRS expression level (*P* < 0.01) and the phosphorylation state ratios of 4E-BP1 (*P* < 0.01), p70S6K (*P* < 0.01), and mTOR (*P* < 0.01) in RE group were significantly greater. The LeuRS expression (*P* < 0.01) and the phosphorylation state ratios of 4E-BP1 (*P* < 0.01), p70S6K (*P* < 0.01), and mTOR (*P* < 0.01) in RE were also greater than AE (Figure 3).

## Discussion

In the current study, we observed a significant increase in the CSA and diameter of gastrocnemius muscle fibers, gastrocnemius wet weight, muscle protein concentration, the phosphorylation state of mTOR signaling, and the relative expression of LeuRS in resistance exercise-trained rats. Aerobic exercise only resulted in a significant but modest increase of muscle protein concentration and p70S6K phosphorylation, and no differences in Sestrin2 expression level between groups was detected. We suggest that LeuRS increases in the RE group only, is part of a coordinated response of the resistance exercised muscle, which may contribute for muscle protein accretion.

We demonstrated for the first time that up-regulation of LeuRS expression and mTOR activation (in its two branches, namely 4EBP-1 and P70S6K) was induced by 4 weeks resistance exercise. In contrast, aerobic exercise training did not increase LeuRS levels, but P70S6K phosphorylation was increased compared to the control group (which suggests a moderate activation compatible with the smaller increases of muscle mass in this group). Since mTOR was not activated in this group, it is possible to suppose that P70S6K activation was caused by other pathways involved in muscle protein synthesis, like GSK3-β. However, the non-activation of mTOR or increased LeuRS in the AE group, suggests a role of LeuRS in activating the mTOR signaling in the RE group.

The effects of LeuRS on protein synthesis in skeletal muscle have not been widely reported, and only 4 studies provide data concerning the role of LeuRS in C2C12 myoblasts or muscle samples. Sato's work showed that LeuRS knockdown via siRNA did not decrease phosphorylated mTOR in differentiated myotubes, nor did it affect hypertrophy (Sato et al., 2018). Son et al. found that LeuRS negatively regulated myoblast differentiation *in vitro*, and this function was independent of its regulation of protein synthesis (Son et al., 2019). Carlin and colleagues found that acute essential amino acid ingestion did not alter the expression of LeuRS in skeletal muscle of young participants, but did up-regulate RagB expression (Carlin et al., 2014). While the consumption of a diet containing twice recommended daily allowance of protein intake for 10 weeks showed a tendency to decrease the mRNA expression of LeuRS, it did not affect the fasting mTORC1 signaling. However, increased total RPS6 might still suggest improved muscular translational capacity to maintain muscle mass (Zeng et al., 2019). Taking these clues into consideration, this might suggest a compensatory system for sensing leucine through Sestrin2. As the affinity of LeuRS for leucine is similar to that of Sestrin2, it remains elusive how these crucial sensors orchestrate leucine sensing to stimulate mTOR activation. In the present work, our results demonstrate the potential of LeuRS to modulate mTOR activity in skeletal muscle. It's reasonable to hypothesize that LeuRS may be a key sensor responding to anabolic signals, and capable of regulating protein synthesis via mTORC1 signaling in skeletal muscle. Moreover, increased muscle protein synthesis is always linked to increased intramuscular availability of amino acids (especially leucine). Thus, it's also reasonable to speculate that resistance exercise training may have resulted in an increased uptake of leucine, and then the increased intramuscular leucine level promotes muscular leucine-sensing by up-regulating of LeuRS expression. However, further studies exploring the relationships between different exercise protocols, age of subjects and LeuRS/Sestrin2-mTOR axis are warranted.

In terms of Sestrin2, studies that have compared untrained young and old mice have reported conflicting results, with one study showing lower levels (Lenhare et al., 2017), another showing increased levels in older mice (Xia et al., 2017b), and a third showing no difference between young and old human males (Zeng et al., 2018). Sestrin2 functions as a negative regulator of mTORC1 (Wolfson et al., 2016), but if Sestrin2 and LeuRS regulate the downstream mTORC1 signaling cooperatively in aging skeletal muscle, it is unclear how they affect mTORC1 activity. In light of these information, some scholars proposed that LeuRS and Sestrin2 may function at different developmental stages (Sato et al., 2018). Results concerning the effects of exercise on the expression level of Sestrin2 are unclear. In the research conducted by Crisol and colleagues, a decrease in basal Sestrin2 in the quadriceps was found in mice following a 4 week chronic exercise protocol on a treadmill running at 60% of peak workload (5 days/week, 60 min/day) (Crisol et al., 2018). However, a series of research from Fu's lab showed a significant increase in Sestrin2 expression in the quadriceps muscle of mice after 6 or 8 weeks treadmill running with progressively increasing workloads (up 75% VO2max) (Liu et al., 2015; Wang et al., 2018; Yu et al., 2019). As for the effects of resistance exercise, to our knowledge only one report exists. Zeng et al. showed no significant change in the expression of Sestrin2 in muscle biopsies from the vastus lateralis following a 12 week lower body strength training protocol (2 days/week, separated by 72 h) (Zeng et al., 2017). On the other hand, we found that 4 weeks of concurrent training (3 days of resistance exercise and 3 days of aerobic exercise weekly) in 2-year old mice lowered Sestrin2 compared to 2-year old controls, but was still significantly greater than young controls. Our results in the present study are in line with Zeng and colleagues' findings concerning the effects of resistance exercise, but being different from the reports of Crisol and colleagues as well as Fu's group concerning the effects of aerobic exercise. In our humble opinion, the differences observed between these reports could be explained by differences in exercise protocols, especially the intensity and duration of exercise sessions. Four weeks of lower intensity of aerobic exercise (<60% VO2max) might not been strong enough to result in an adaptive change of Sestrin2 expression, while higher intensity and longer duration might lead to possible discrepancies in the results (such as 60 vs. 75% VO2max; 4 weeks training vs. 6–8 weeks training).

Some limitations should be taken into consideration when interpreting the results of this study. First, we did not investigate the effects of acute exercise and time course changes on the expression of leucine-sensing related proteins (i.e., LeuRS, Sestrin2 and mTORC1 signaling). This information may help explain the seemingly contradictory results between gastrocnemius muscle protein concentration and the activity of mTOR signaling in aerobic exercise-trained rats. Second, we were not able to use a group with RagGTPases knockout mice, to fully establish the interaction between LeuRS, Sestrin2, RagGTPases, and downstream mTORC1 signaling.

## Conclusion

In conclusion, 4 weeks resistance exercise training induced favorable adaptations in the gastrocnemius muscle of young rats, including increased wet weight, protein concentration, muscle diameter and CSA, while aerobic exercise only resulted in a significant increase of protein concentration. These positive results were likely the result of elevated leucine-sensing and subsequent protein deposition. From a molecular mechanistic standpoint, LeuRS/mTOR signaling axis may be involved in this adaptive process induced by exercise (especially resistance exercise), as no differences between groups in Sestrin2 were found. Further studies are needed to investigate these hypotheses via the use of bioinformatics or gene chip assay, and should be further verified by using targeted gene knockout/overexpression animal models or blockers/agonists (i.e., LeuRS and mTOR). Future studies are also needed to determine whether these adaptations translate into healthy benefits in human subjects when interventions are performed with different protocols concerning the exercise duration and the intensity.

## Data Availability Statement

The raw data supporting the conclusions of this article will be made available by the authors, without undue reservation.

## Ethics Statement

The animal study was reviewed and approved by Animal Ethics Committee of Jinggangshan University and Chengdu Sport University.

## Author Contributions

QW, NZ, and ZX designed the experiment. YZ, HS, YY, XD, and SL performed the experimental analyses and collected the data. YZ, JC, and HS performed the statistical analyses. YZ and HS wrote the manuscript. JC, QW, NZ, and ZX proofread the manuscript. All authors read and approved the final version.

## Funding

This study was supported by grants from the National Natural Science Foundation of China (Nos. 31960192 and 31900842), the Jiangxi Provincial Science Fund for Distinguished Young Scholars (No. 20202ACBL216004), and the Jiangxi Provincial Natural Science Foundation (No. 20192BAB205081).

## Conflict of Interest

The authors declare that the research was conducted in the absence of any commercial or financial relationships that could be construed as a potential conflict of interest.

## Publisher's Note

All claims expressed in this article are solely those of the authors and do not necessarily represent those of their affiliated organizations, or those of the publisher, the editors and the reviewers. Any product that may be evaluated in this article, or claim that may be made by its manufacturer, is not guaranteed or endorsed by the publisher.
